# Supplementary material for: Human birth tissue products as a non-opioid medicine to inhibit post-surgical pain
Source: eLife. 2024 Dec 13;13:RP101269. doi: 10.7554/eLife.101269 (PMC11643635; doi:10.7554/eLife.101269)
Supplement: Figure 3—figure supplement 1—source data 1. [file elife-101269-fig3-figsupp1-data1.pdf]

Full unedited gel for Figure 3-figure supplement 1A

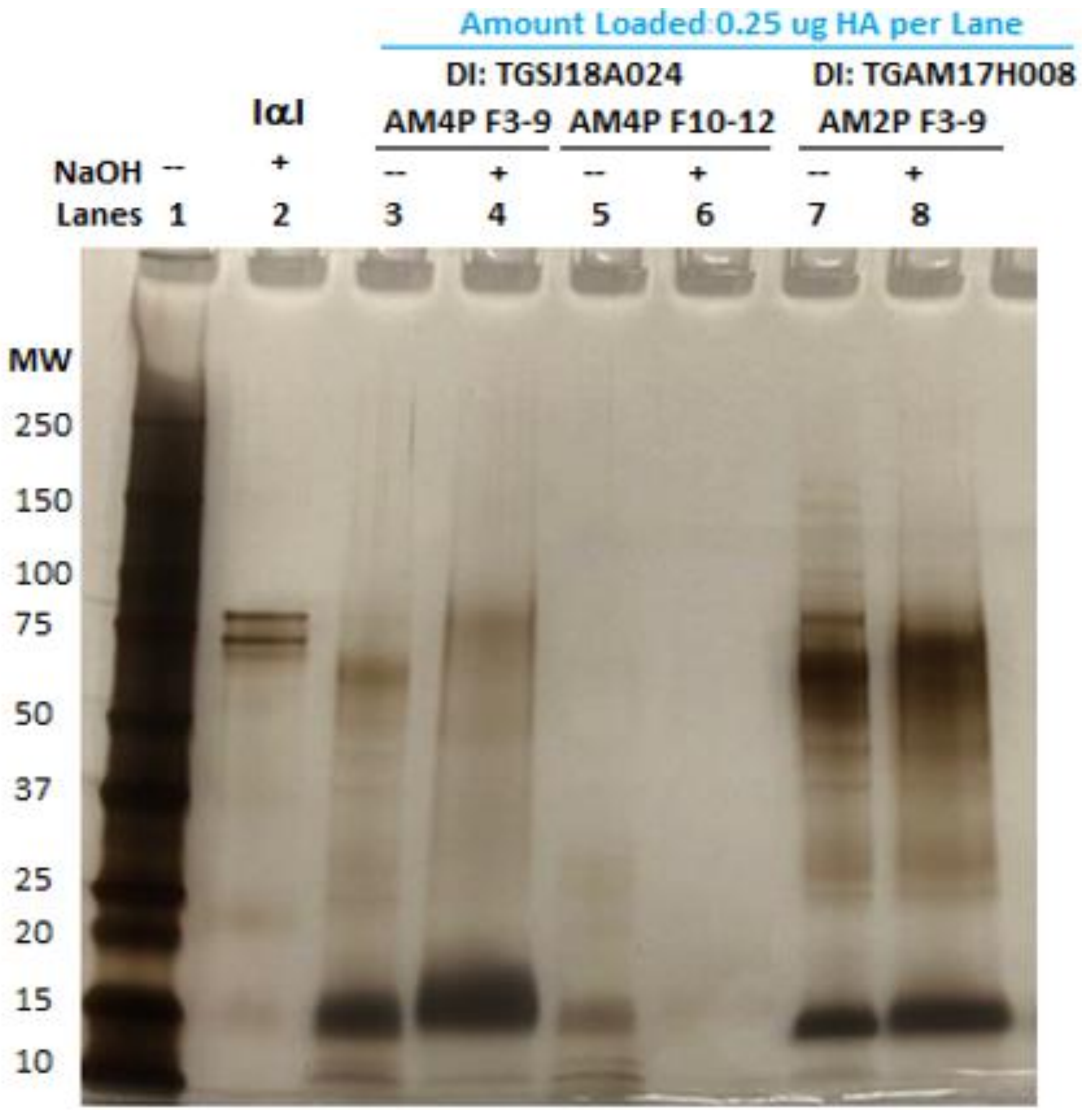

Lanes 1 – 4, 7,8 are shown in Fig. S4A. Lanes 5, 6 is cut off because the data is not included in the manuscript.

Full unedited gel for Figure 3-figure supplement 1B

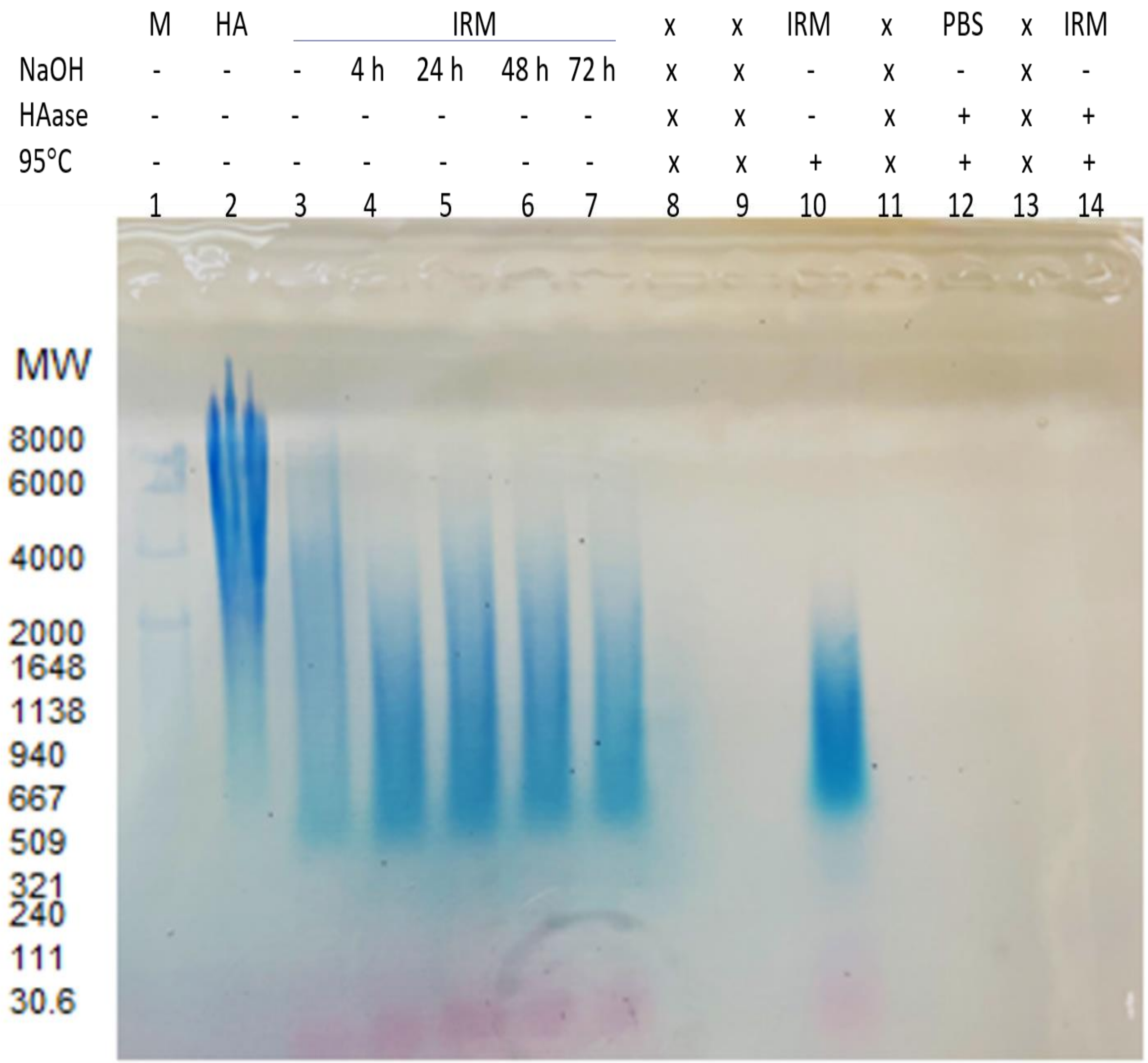

Lanes 1 – 3 are shown in Fig. S4B. Lanes 4 – 14 is cut off because the data is not included in the manuscript.

Full unedited gel for Figure 3-figure supplement 1C

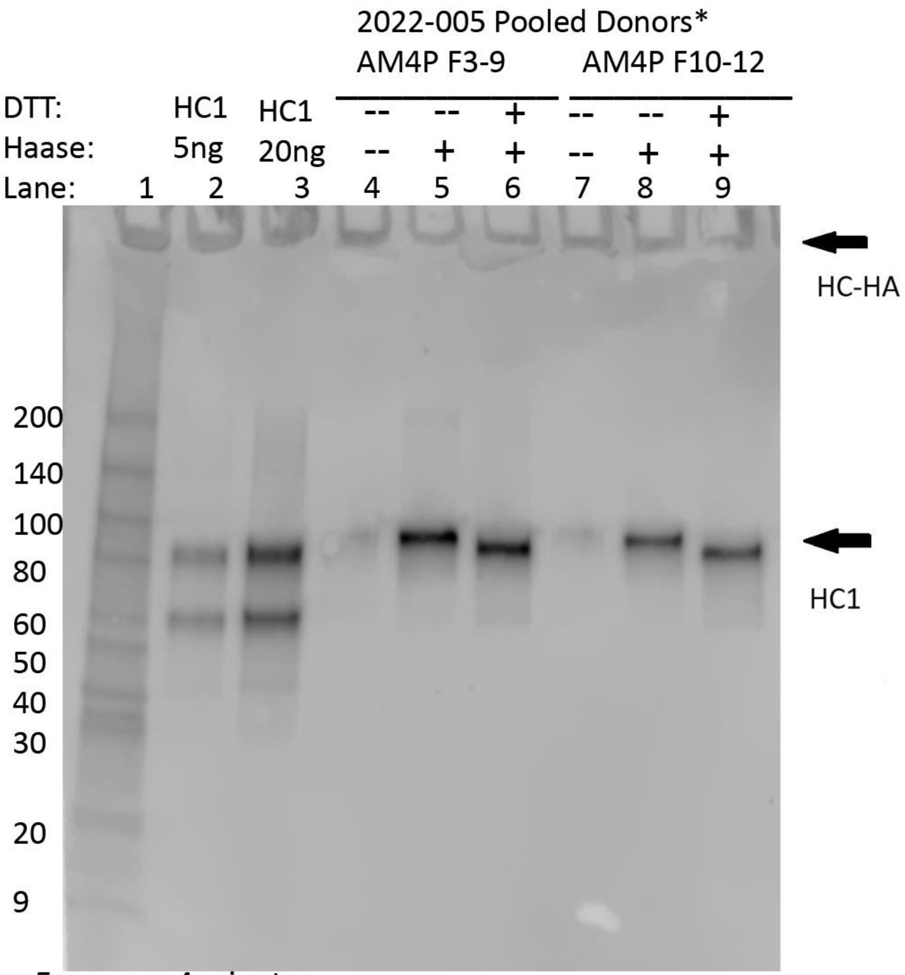

Exposure: 4 minutes

*Pooled Donors: TGBK2022CO18, TGRR22C035, TGSM22C009 TGBA22C022, TXM22C013, TGJX22C021, TGBA22C023

Lanes 1 – 6 are shown in Fig. S4C. Lanes 7 – 9 is cut off because the data of AM4P F10-12 is not included in the manuscript.

Full unedited gel for Figure 3-figure supplement 1D

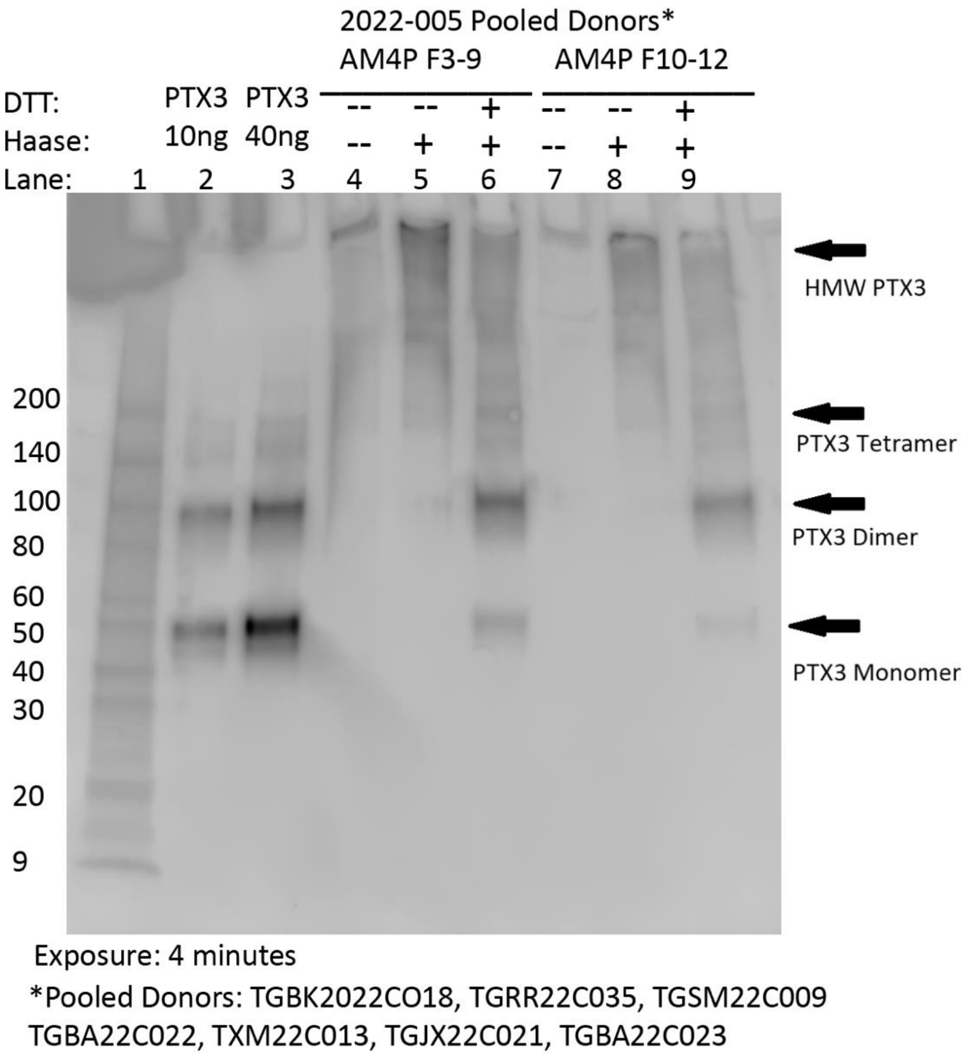

Lanes 1 – 6 are shown in Fig. S4D. Lanes 7 – 9 is cut off because the data of AM4P F10-12 is not included in the manuscript.
