# Supplementary figures and images for: Human birth tissue products as a non-opioid medicine to inhibit post-surgical pain
Source: eLife. 2024 Dec 13;13:RP101269. doi: 10.7554/eLife.101269 (PMC11643635; doi:10.7554/eLife.101269)

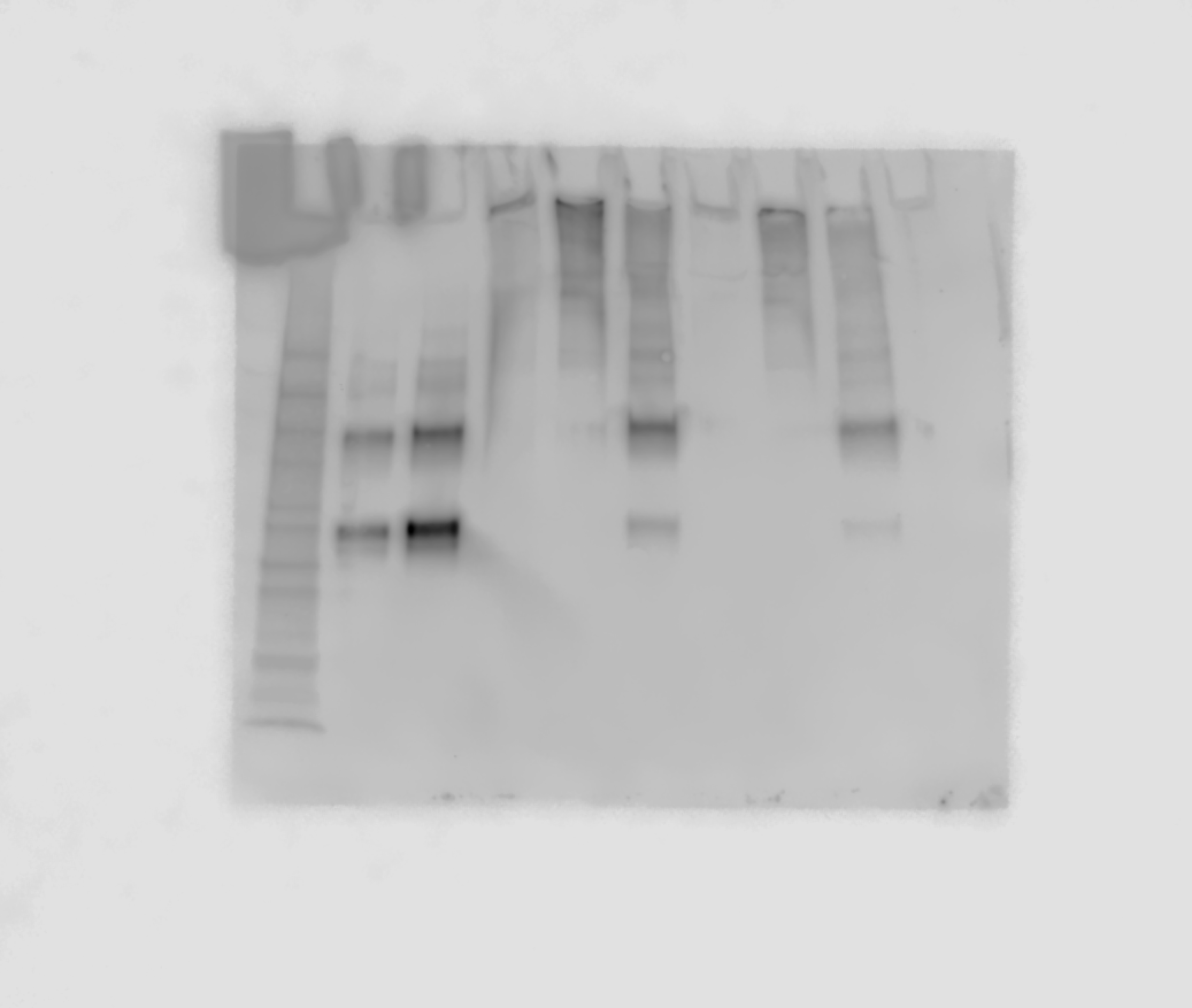

Supplement: Figure 3—figure supplement 1—source data 2. [file elife-101269-fig3-figsupp1-data2.zip › Figure 3-figure supplement 1_Source Data 2/Figure 3-figure supplemental 1D.tif]

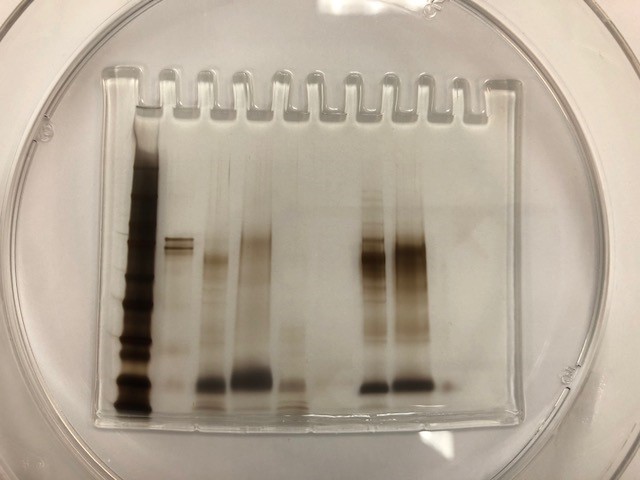

Supplement: Figure 3—figure supplement 1—source data 2. [file elife-101269-fig3-figsupp1-data2.zip › Figure 3-figure supplement 1_Source Data 2/Figure 3-figure supplement 1A.jpg]

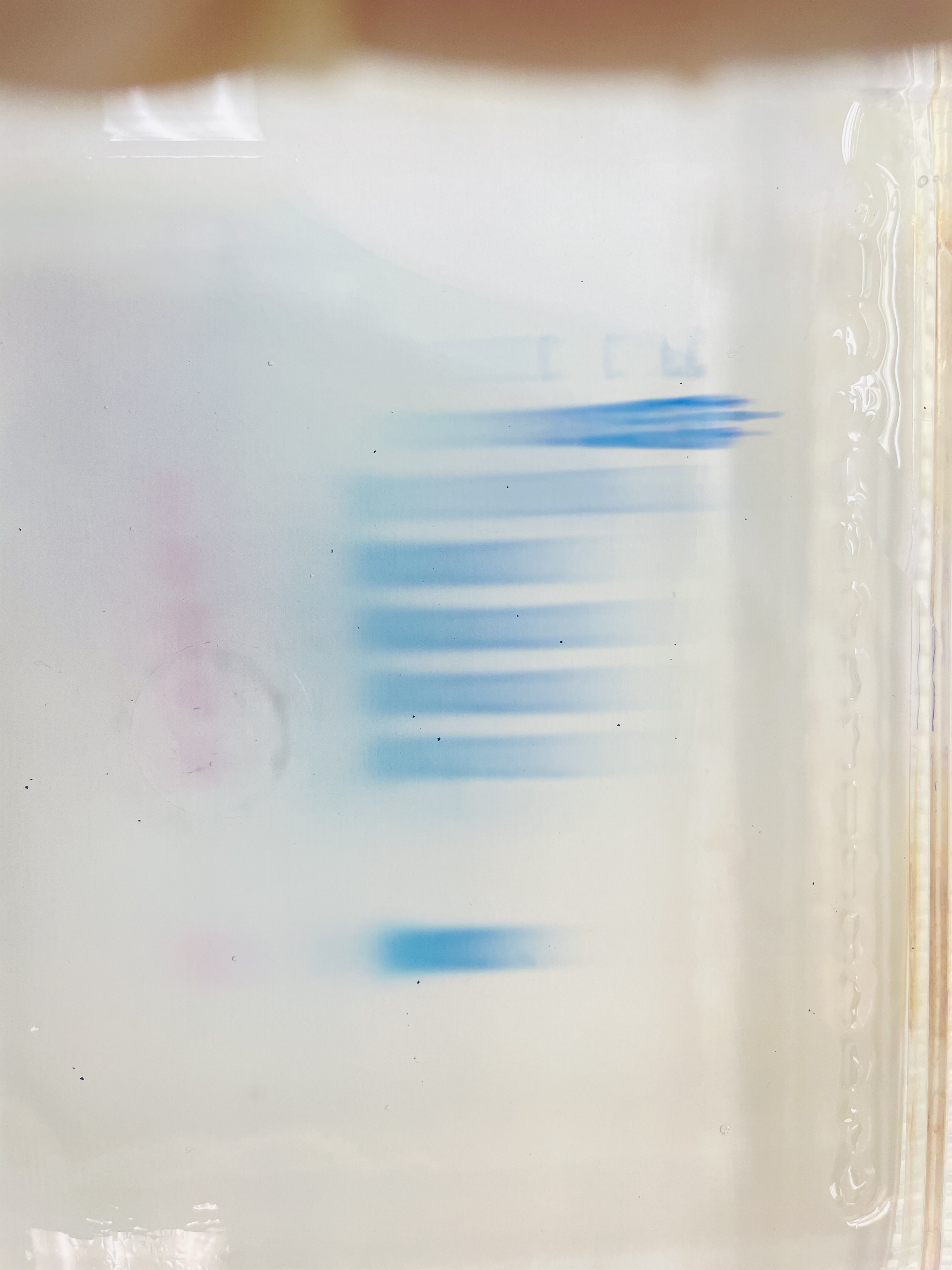

Supplement: Figure 3—figure supplement 1—source data 2. [file elife-101269-fig3-figsupp1-data2.zip › Figure 3-figure supplement 1_Source Data 2/Figure 3-figure supplement 1B.jpg]

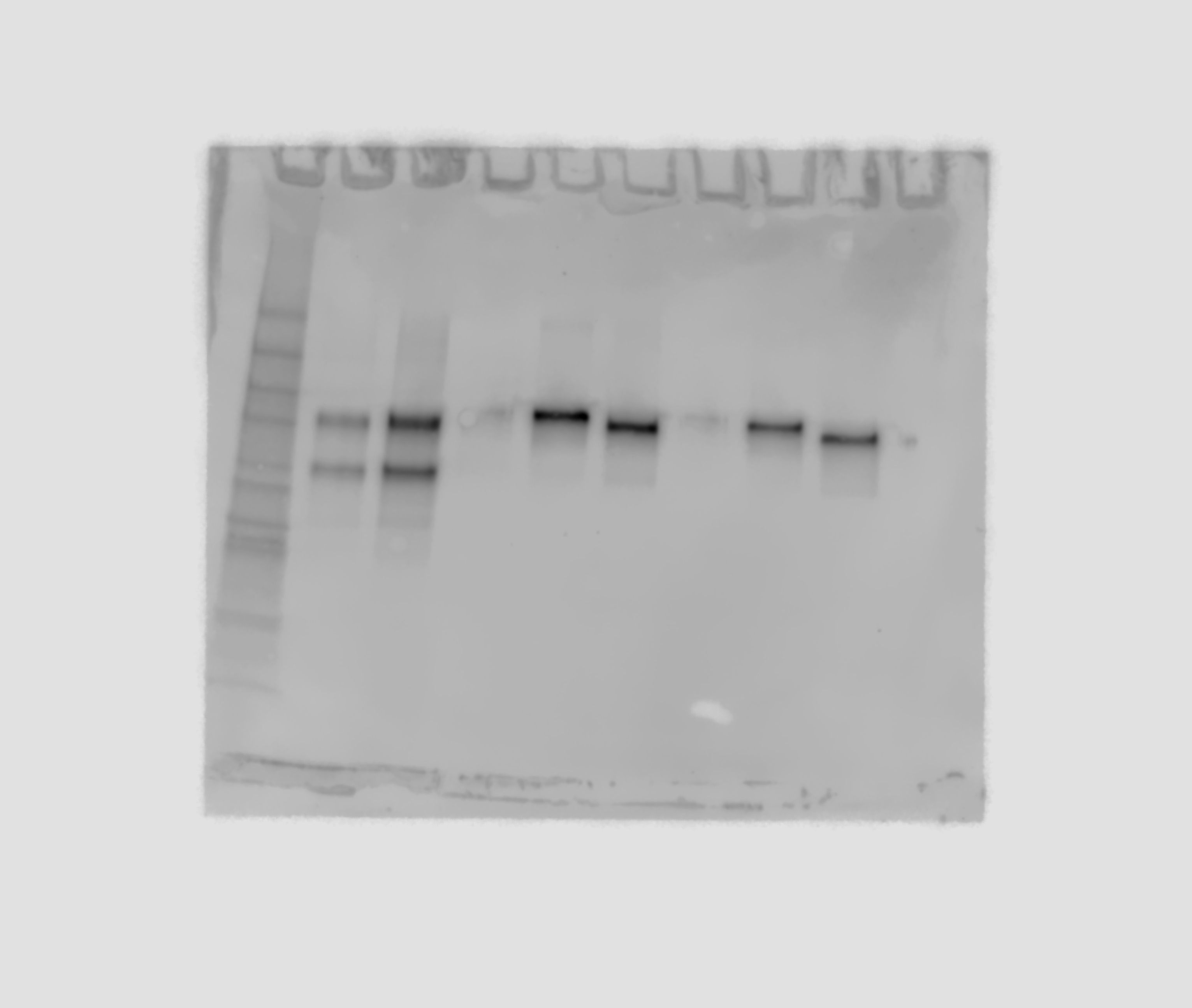

Supplement: Figure 3—figure supplement 1—source data 2. [file elife-101269-fig3-figsupp1-data2.zip › Figure 3-figure supplement 1_Source Data 2/Figure 3-figure supplement 1C.tif]

# Full unedited gel for Figure 4-figure supplement 1B-CD44

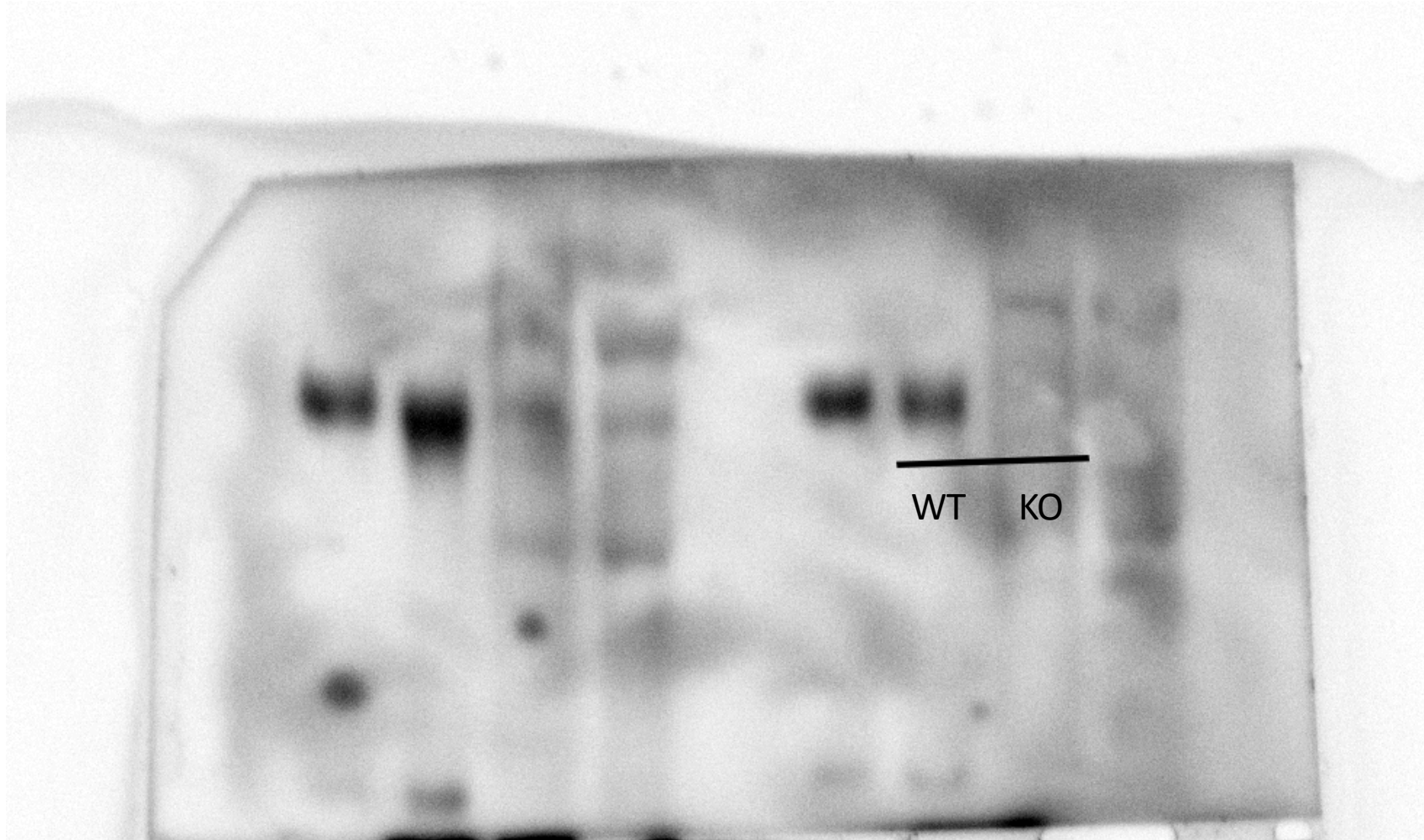

Supplement: Figure 4—figure supplement 1—source data 1. [file elife-101269-fig4-figsupp1-data1.pdf]

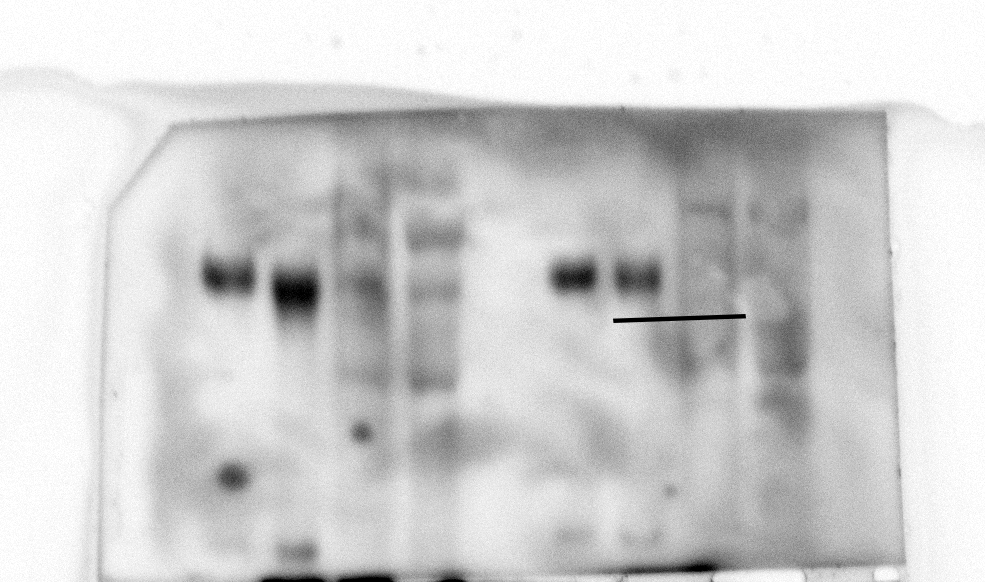

Supplement: Figure 4—figure supplement 1—source data 2. [file elife-101269-fig4-figsupp1-data2.zip › Figure 4-figure supplement 1_Source Data 2/Figure 4-figure supplement 1B.tif]
