# Supplementary material for: Human birth tissue products as a non-opioid medicine to inhibit post-surgical pain
Source: eLife. 2024 Dec 13;13:RP101269. doi: 10.7554/eLife.101269 (PMC11643635; doi:10.7554/eLife.101269)
Supplement: Supplementary file 1. — Knocking out of CD44 did not significantly alter the intrinsic membrane property of DRG neurons, as compared to that in WT mice. [file elife-101269-supp1.docx]

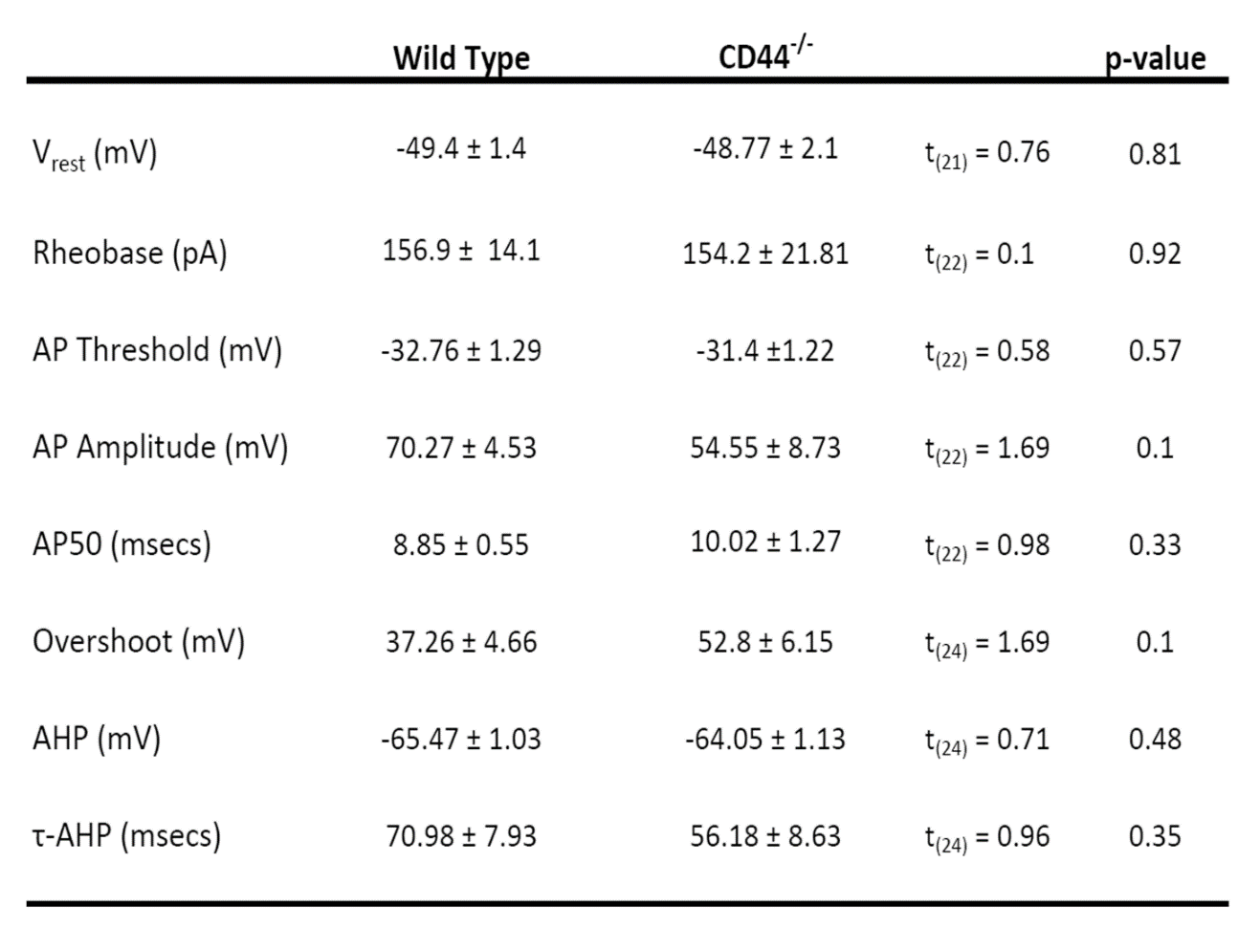


**Supplementary File 1. The measures of intrinsic membrane properties of small-diameter DRG neurons in WT and CD44 KO mice.** Knocking out of CD44 did not significantly alter the intrinsic membrane property of DRG neurons, as compared to that in WT mice.
